# Study on the Properties of PLA- and PP-Based Films for Food Applications Incorporating Orange Peel Extract from Agricultural by-Products

**DOI:** 10.3390/polym16091245

**Published:** 2024-04-29

**Authors:** Ana Maria Tone, Nuria Herranz Solana, Muhammad Rehan Khan, Angela Borriello, Elena Torrieri, Carmen Sánchez Reig, F. María Monedero Prieto

**Affiliations:** 1 Packaging, Transport & Logistics Research Center (ITENE), Albert Einstein 1, 46980 Paterna, Valencia, Spain; anamaria.tone@itene.com (A.M.T.); nuria.herranz@itene.com (N.H.S.); csanchez@itene.com (C.S.R.); 2 Department of Agricultural Science, University of Naples Federico II, Via Università 100, 80055 Portici, Italy; muhammadrehan.khan@unina.it (M.R.K.); angela.borriello@unina.it (A.B.)

**Keywords:** active packaging, antimicrobial activity, antioxidant activity, polylactic acid, extrusion, orange peel extract, food loss, food contact material, NIAS, migration

## Abstract

The aim of this work was to develop active packaging based on polypropylene (PP) and polylactic acid (PLA) matrices using a high value by-product extracted from orange peel as an active compound for food packaging applications. Different films with and without orange peel extract (OPE) based on PP and PLA were obtained via cast extrusion and characterized in terms of their mechanical, thermal, optical, and sealing properties. The films obtained were transparent, but when OPE was incorporated, the transmittance spectrum decreased, causing slight coloration. Mechanical properties were affected by the incorporation of OPE, as elongation at break and tensile strength increased in the cross-direction of the PP film, although the main differences found were related to the polymer itself. In addition, sealing strength also increased via the incorporation of OPE in the PP matrix. However, thermal properties were not affected by OPE in the PP matrix but slightly decreased stability in PLA. Regarding antimicrobial activity in in vitro studies, no inhibition of the growth of *Listeria innocua*, *Saccharomyces cerevisiae*, *Aspergillus niger*, or *Escherichia coli* was observed. Finally, antioxidant activity was observed in in vitro studies with 2,2-Diphenyl-1picrylhydrazyl (DPPH) radical. The results of this study showed that the obtention of materials with OPE incorporated into the PLA and PP matrix is feasible. The new materials obtained can be used for applications of oxidation-sensitive fresh products.

## 1. Introduction

Both food waste and the generation of food by-products are serious problems for society, causing a negative environmental impact, while undermining the efficiency of the productive sector and its competitiveness. According to the Food and Agriculture Organization of the United Nations (FAO), the food that is lost and wasted could feed 1.26 billion people every year [1].

In recent years, by-product recovery has received great interest as a tool with which to achieve a circular economy in terms of waste reduction and efficient waste management [2]. Directive 2008/98/EC is a legal framework for waste treatment in the EU [3]. Its main objective is achieving a reduction in waste production following a waste hierarchy model consisting of different steps: the first step consists of waste prevention through proper waste management, the second step consists of the reuse of by-products to minimize or, if possible, avoid their release into the environment, the next steps are the recycling route and recovery strategies, and the final step is disposal [4]. Focusing on the fruit and vegetable sector, production in the EU in 2022 was estimated to be worth EUR 7.34 billion, representing more than 14% of all agricultural production [5]. Orange peel (OP) represents almost 60% of the by-products of juice processing. This by-product is rich in flavonoids, phenolic acids, carotenoids, essential oils, lignin, cellulose, and hemicellulose [6]. On the other hand, it is well known that by-products obtained during food processing, such as the above-mentioned orange peel, are underutilized despite the nutritional and bioactive richness present, with a wide spectrum of biological functions. Therefore, valorizing these fruit by-products represents a solution towards achieving more sustainable agri-food systems. One example would be using them to develop active food packaging materials capable of preserving the quality of perishable foods and reducing food waste [7].

Active packaging refers to a type of packaging system designed to interact actively with the product it contains by releasing or absorbing substances into or from the packaged food or the environment surrounding the food with the final objective of extending the food’s shelf life [8]. Active packaging can be tailored to have many functions, such as, antimicrobial, antioxidant, insect-repellent, ethylene-absorbing, oxygen-absorbing, etc. Its designed function will depend upon the main mechanisms involved in the deterioration of the product to be packed.

Over the last few years, different active packaging solutions with antimicrobial or antioxidant properties for extending the shelf life of perishable food products have been developed by using synthetic or natural bioactive compounds [9,10]. The active substances can be introduced directly into the polymeric matrix in bulk or can be added to filmogenic formulations and applied via coating techniques. In addition, to protect the active ingredients, which could degrade or volatilize during processing, they are often encapsulated to increase their thermal stability and thus their retention in the final material [11]. Recently, it has been shown that bioactive extracts of orange peel residues can be used to produce active films with antioxidant [12] and antimicrobial properties when combined with avocado seed extract [13]. In addition, bitter orange peel extract has been used to develop new bioactive nanofibers based on ethyl cellulose (EC) and soy protein isolate (SPI), with antioxidant and antimicrobial properties [14]. On the other hand, Casas et al. (2022) [15] applied the scCO_2_ extraction and sequential fractionation technique to orange peel to obtain active substances rich in bioactive compounds with strong antioxidant and antimicrobial activity potentially useful in the development of active packaging. In this sense, Casas et al. (2022) [15] impregnated polypropylene with orange peel extracts, obtained via supercritical fluid extraction, in a satisfactory way for the improvement of food preservation.

Active compounds have been incorporated into conventional materials such as polyolefins, e.g., low-density polyethylene (LDPE) and linear low-density polyethylene (LLDPE) PP, as well as in biopolymers such as PLA or edible films. Recently, Vidal et al. (2023) [16] developed a packaging material with controlled release of ethyl lauroyl arginate (LAE). This compound is used in the food industry for protecting dairy and meat products. The active material consisted of a biodegradable trilayer structure composed of extruded PLA film followed by an active electrospun PLA mat and a coating with chitosan. Also, Cerro et al. (2021) [17] impregnated lignin nanoparticles with cinnamaldehyde and incorporated them into biodegradable materials based on PLA by means of supercritical impregnation for packaging and biomedical applications. Wang and Rhim (2016) [18], on the other hand, incorporated grapefruit seed extract into LDPE and PLA blended with thermoplastic starch (TPS) to obtain antimicrobial films, the antimicrobial activity of which was tested against the food-borne pathogens *L. monocytogenes* and *E. coli*, with promising results as the films exhibited clear antibacterial activity.

Active materials, in addition to intentionally releasing substances into food or its environment, can also transfer substances unintentionally. In the latter case, we refer to migration, which can occur due to the presence of additives, residual monomers, impurities that come with raw materials, contamination, etc., which are, in essence, migrant substances. All of this should be taken into account when assessing the safety of these materials for food contact [19,20,21,22]. In plastic food contact materials (FCMs), migration can occur from the inner side of the packaging and the internal layers due the diffusion processes where a movement of molecular structures from the high-concentration to the low-concentration region occurs through a gradient, until equilibrium is reached, as Fick’s second law describes [21]. The migrants from FCMs can be intentionally added substances (IAS) introduced during the production of the FCMs, such as monomers, additives, catalysts, and production aids. Nevertheless, impurities and reaction products such as oligomers, by-products, and degradation products that are non-intentionally added substances (NIAS) in the formulation of the FCMs [22] migrate from the FCMs. Migration depends on several factors, such as the contact surface area between the packaging and the food; the nature of the migrant in terms of molecular weight and volatility, water solubility, and octanol solubility; the FCM type in terms of permeability; the nature of the food in contact with the packaging, for example, high-fat-content foods or hydrophilic foods; and also the storage period of the packaged product and the conditions of its use [20].

The main objective of this work is to develop active packaging materials for food contact incorporating agricultural by-products in conventional polymers (from fossil sources) and bio-based polymers. The development of this type of material presents characteristics that make it unique and interesting from different angles, in line with the circular economy. Firstly, they incorporate a by-product of the fruit and vegetable industry, which allows for its valorization by reintroducing it into the production chain. On the other hand, this study explores the use of conventional polymeric matrices, such as PP, and bio-based polymers, such as PLA, as vehicles for the active ingredient. Finally, the fact that they are active films makes them interesting for extending the shelf life of foodstuffs, allowing a reduction in food losses.

In this sense, in the study, OPE was incorporated into two different polymeric matrices, PP and PLA. In the conventional polymer, it was introduced at a concentration of 10% (*w*/*w*), and in the PLA, it was incorporated at a concentration of 7.5% (*w*/*w*). Once the films were obtained, they were characterized on the basis of their physical–mechanical, thermal, and optical properties. Additionally, their antimicrobial and antioxidant properties were studied, as was their safety from the food contact point of view.

## 2. Materials and Methods

### 2.1. Materials

PP-grade PR230C1E was purchased from Ibiplast (Alicante, Spain), and PLA-grade Luminy^®^ LX175 was purchased from Total Energies Corbion (Oss, The Netherlands). DPPH was purchased from Sigma-Aldrich (Milan, Italy). The active ingredient, OPE, was provided by Bio Base Europe Pilot Plant (BBEPP, Gent, Belgium).

### 2.2. Characterization of the Active Ingredient

The active ingredient, OPE, was characterized via HPLC-ESI-TOF-MS, in accordance with the method described by Razola-Díaz et al. (2021) [23].

### 2.3. Films Preparation

For the processing of the films, in the first stage, two active compounds were prepared, both consisting of a blend of the virgin polymer (PP or PLA) and 10% (*w*/*w*) OPE for the PP blend, or 7.5% (*w*/*w*) OPE for the PLA blend. For this purpose, a ZSK 26K twin-screw extruder (Coperion, Stuttgart, Germany) with L/D 40 was used. The OPE was incorporated through the liquid port, and the virgin polymer was incorporated into the dosing hopper and mixed with the OPE in accordance with the specified theoretical concentrations. The temperature profiles set along the 9-barrel zones were 140/180/190/190/190/195/195/195/195 (°C) for the formulation of PP compounds incorporating OPE (PP + OPE) and 190/195/200/200/200/200/200/200/200 (°C) for the PLA formulation incorporating OPE (PLA + OPE). As a result, a thread measuring about 2 mm in diameter was obtained and passed through a cold-water bath to solidify. Finally, it was pelletized into small pieces of approximately 3 mm length. PP = based pellets were obtained with an extrusion speed of 1000 rpm and a throughput of 10.8 kg/h, and for PLA-based pellets, an extrusion speed of 1100 rpm and a throughput of 16 kg/h were used.

In the second stage, bilayer films (the references and structure are shown in Table 1) were processed with a co-extrusion line, Dr. Collin MF-EXB-600 (Ebersberg, Germany), based on three single co-extruders (E30P 25L/D). In this second stage, to obtain the bilayer films, extruders were fed the pellets and active pellets obtained in the first step of the production process. Extruders B and C were used to manufacture a two-layer structure (BC) of PP and PP-OPE. The processing width was 360 mm, and a production speed of 5 m/min was set for all the film references produced. The temperature profile set for extruder B was 220/230/230/235/240, and for extruder C, it was 200/235/225/230/235 (°C). On the other hand, for PLA, extruders A and B were used to manufacture the two-layer structure of PLA-OPE. The temperature profiles that were set were 165/170/175/180/185 (°C) for extruder A, and 170/210/215/220/220 (°C) for extruder B.

The bilayer active films obtained presented OPE on the food contact side. Bilayer structures of PP/PP and PLA/PLA as controls were obtained as were active films PP/PP-OPE and PLA/PLA-OPE with a thickness relation of 25 µm/50 µm in all cases.

### 2.4. Film Characterization

#### 2.4.1. Optical Properties

The optical properties of the films were analyzed via spectrophotometry. UV-VIS spectra were recorded by using a spectrophotometer, JASCO UV 630 (Madrid, Spain), at wavelengths from 200 to 900 nm in the transmittance (%) photometric mode. In addition, a visual inspection was carried out.

#### 2.4.2. Thickness and Grammage

The thickness of the films was determined by using a digital micrometer, IS-S112B (Mitutoyo, Japan).

The grammage of the films was determined by accurately weighing pieces of film with a surface area of 12 cm^2^, cut using a calibrated template.

#### 2.4.3. Mechanical Properties

Mechanical properties were analyzed through the parameters tensile strength (TS), elongation at break (EB), and Young’s modulus (YM) following the ISO 527-3:2018 standard [24]. These mechanical parameters were measured at room temperature by using a Testometric machine (M350–20 CT, Testometric Co., Ltd., Lancashire, UK). Tests were performed using rectangular probes (10 cm length × 1.5 cm width) and with a crosshead speed of 200 mm·min^−1^. Before testing, all samples were equilibrated for 16 h at 50% RH. Six repetitions were performed for each formulation. Measurements were taken in the longitudinal and cross-direction.

#### 2.4.4. Sealability

Sealing trials were performed with HSE-3 Gradient Laboratory Heat Sealer, RDM (Hertfordshire, UK) test equipment. Different sealing temperatures were evaluated. Good sealing was considered when the 2 films tested could not be manually separated. In addition, once sealed, the sealing strength of the films was evaluated by using a Testometric machine (M350–20 CT, Testometric Co., Ltd., Lancashire, UK), measuring the force required to open the sealing area.

#### 2.4.5. Thermal Analysis

The thermal stability of the samples was determined by using TGA Q-5000 from TA-Instruments (New Castle, DE, USA), with a ramp of 20.00 °C·min^−1^ from 50 °C to 500 °C, a flow rate of 25 mL per minute of dry nitrogen, and isothermal conditions for 1.00 min, analyzing samples with a mass lower than 10 mg.

A differential scanning calorimeter (DSC Q200, TA Instruments, USA) was used to investigate the thermal properties of the film. Each sample (≈6 mg) was placed in an aluminum pan and hermetically sealed. The film samples were cooled to −10 °C, heated (10 °C·min^−1^) from −10 °C to 200 °C, and equilibrated for 1 min. After equilibration, they were scanned (10 °C·min^−1^) from 200 °C to −10 °C. Data were analyzed using TA Universal Analysis software (version 5.5.22). The maximum degradation temperature (T_max_) was determined from the maximum temperature of the peak in the thermogram curve; this parameter represents the maximum weight loss. In addition, onset temperature (T_onset_), representing the point in the thermogram where a deflection was first observed from the established baseline, prior to the thermal event, was determined. The residue was also determined. This parameter is defined as the % of material that remains at the end of the test (once the temperature exceeds 500 °C).

From the DSC curves, the following parameters were determined: glass transition on-set (T_on-set_), end-set (T _end-set_), and inflection point (T_inflection point_) temperatures; cold crystallization on-set (T_on-set_), end-set (T_end-set_), and peak (T_pk_) temperatures and enthalpy (ΔH); melting on-set (T_on-set_), end-set (T_end-set_), and peak (T_pk_) temperatures and enthalpy (ΔH).

#### 2.4.6. Antimicrobial Activity

The antimicrobial activity of the developed films was evaluated via two procedures. On the one hand, their activity in the vapor phase was examined. This test evaluated whether or not the films showed activity in the headspace of the package, simulating the conditions most commonly found in fruit and vegetable packages. On the other hand, to analyze whether or not the films present functionality through another type of mechanism, their activity was analyzed by means of a procedure involving intimate contact. In this way, contact with packaged foods where this type of contact occurs was simulated.

Antimicrobial activity in the vapor phase

To evaluate the efficacity of the active substances against microorganisms in the vapor phase, a representative of each of the following groups of microorganisms was selected to carry out the evaluation of the antimicrobial activity of the samples. In this sense, *Escherichia coli* was employed to cover Gram-negative bacteria, *Listeria innocua* was used for Gram-positive bacteria, *Saccharomyces cerevisiae* was used for yeasts, and finally, *Aspergillus niger* was chosen as a mold representative. *Escherichia coli* (CETC 516), *Saccharomyces cerevisiae* (CECT 1383), *Aspergillus niger* (CECT 2807), and *Listeria innocua* (CECT 910) were obtained from Colección Española de Cultivos tipo (CECT, Valencia, Spain). Then, the film samples were cut to obtain a circular area measuring 9 cm^2^ to cover the whole surface of the Petri dish lid.

Petri dishes were seeded by using an automatic sowing machine, easySpiral Pro^®^ (Interscience, Saint Nom la Bretèche, France), applying a constant volume of 50 µL of the inoculum. For all the microorganisms tested, the working inoculum was established to have between 2.5 × 10^4^ and 10 × 10^4^ cfu/mL of microorganisms. Once the Petri dishes were seeded, the films were adhered to the inner part of the lids, and they were sealed with parafilm^®^. Finally, the Petri dishes were incubated in the optimal growth conditions for each one of the selected microorganisms for 24 h at 36 °C for *E. coli* and *L. innocua*, and for 3 days and 5 days at 25 °C for *S. cerevisiae* and *A. niger*, respectively. All samples were tested in triplicate.

Once the incubation time finished, the effectiveness of the samples was analyzed. For this, the plates were visually evaluated, and the diameter of the inhibition zone was measured in each one. Then, the percentage of inhibition was calculated by using Equation (1):

(1) $$\%\;inhibition=\frac{Area\;inhibition\;zone}{Area\;of\;the\;petri\;dish}\cdot 100$$

Antimicrobial activity via direct contact

To analyze the activity of the films in direct contact with the food, an internal protocol based on the standard UNE-EN 1104-2019 paper and board for contact with foodstuffs—the determination of the transfer of antimicrobial constituents [25]—was used. For this, circular pieces of film with a diameter of 15 mm were cut out and put into contact with an inoculated plate with the same four microorganisms used in the vapor phase test. The side of the film put into contact with the inoculated agar was the food contact side. Three pieces of film were placed on each inoculated plate, and three Petri dishes per microorganism were subjected to the test. Simultaneously, inoculated plates without film samples were prepared as positive controls and non-inoculated plates without the sample specimens (just agar) were prepared as negative controls.

After that, the inoculated plates in contact with the circular pieces of films were incubated in the same conditions as those described for the vapor phase test. which differed as a function of the microorganism (as explained for the evaluation of the antimicrobial activity in the vapor phase). After the incubation period, the plates were visually inspected for the presence or absence of inhibition zones. A zone of inhibition is understood as the absence of growth of the inoculated microorganism (translucent zone) in an area at least 2 mm wide at the edges of the tested specimens. In the case that we found inhibition zones, the result would be have been positive, and confirming that the films transferred water-soluble antimicrobial substances to the agar, so it could be stated that they possess antimicrobial activity in direct contact with the evaluated microorganisms.

#### 2.4.7. Antioxidant Activity

The antioxidant activity of the film samples was evaluated by using the DPPH radical scavenging method either via extraction or through direct contact of the films. For direct extraction (from films), the method modified by Jabraili et al. (2021) [26] was used to investigate antioxidant activity. For this, 5 mL of methanol was added to 4 cm^2^ of each film sample, and the mixture was vortexed for 1 min. The supernatant was utilized to assess the inhibitory efficacy following centrifugation for 10 min at 10,000 rpm. A 25 ppm DPPH methanol solution was added to 1 mL of the resultant supernatant. The mixture was incubated for 30 min at room temperature in the dark, and after that, a spectrophotometer was used to measure its absorbance at 517 nm. Using Equation (2), the percentage of DPPH inhibitory potency was then determined:

(2) $$Antioxidant\;activity\;\left(\%\right)=\frac{AC-AS}{AC}\times 100$$

For direct contact (with films), the Priyadarshi, Kim, and Rhim (2021) [27] method was modified to determine the antioxidant activity of the film samples. In a nutshell, 2.5 mg of DPPH was dissolved in 100 mL of methanol to create a methanolic solution. The absorbance was measured at 517 nm after 4 cm^2^ of each film sample was added to 5 mL of methanol (to ensure direct contact) and incubated for 30 min at room temperature in the dark. As a control, the same test was run without using any film samples. Using the same equation as that above, the antioxidant capacity of the film samples was determined as a percentage of DPPH.

#### 2.4.8. Food Safety Assessment

Migration tests

Overall migration tests were carried out in accordance with the provisions of Regulation (EU) No 10/2011 [28] and UNE-EN 1186-3:2023 [29]. The test conditions of time and temperature were 10 days at 40 °C in 10% ethanol (*v*/*v*) (simulant A) and 3% acetic acid (*w*/*v*) (simulant B). These conditions were selected in accordance with Annexes 3 and 5 of Regulation (EU) No 10/2011 [28]. The tests were carried out via one-sided contact, with a surface–volume ratio of 6 dm^2^ of material/kg of food.

The specific migration testing of substances restricted in Annex II of Regulation (EU) No 10/2011 [28], i.e., primary aromatic amines (PAAs) and metals, was carried out in accordance with an in-house methodology and the Plastics Regulation. The exposure conditions were as follows: 10 days at 40 °C. PAAs and metals were determined in simulant B, which is known to be the worst-case scenario for these substances. PAAs were analyzed by using an ultra-performance liquid chromatograph (UPLC Waters Acquity l-Class) coupled to a mass spectrometer with an orthogonal time-of-flight hybrid quadrupole analyzer (QToF-MS), (Waters Corporation, Milford, MA, USA), and metals were determined using ICP-MS iCAP RQ (Thermo Fisher Scientific Madrid, Spain).

Screening of non-intentionally added substances

To carry out the screening of NIAS, the migration extracts of the different references in simulants A and B were analyzed by using different chromatographic techniques to screen and identify substances present in the extracts that migrated to simulants A and B. Specifically, the volatile, semi-volatile, and non-volatile fractions were analyzed.

To determine the volatile substances, 0.5 g of each sample was introduced into headspace vials, sealed, and afterwards incubated at 85 °C for 20 min. Sampling was performed via solid phase micro extraction (SPME) using the 50/30 µm SPME fiber DVB/CAR/PDMS. The headspace content was then directly analyzed by using a Gas chromatograph coupled to a triple quad mass detector (GC-QQQ).

To determine the semi-volatile substances, 100 µL of each migration extract was analyzed by using a gas chromatograph (GC Waters 7890A, Agilent, Santa Clara, CA, USA) coupled with a time-of-flight mass detector (GC-TOF-MS) with a GC SLB-5ms 30 m × 0.25 mm × 0.25 µm column. In addition, the semi-quantification of the substances found was carried out using benzophenone (Sigma-Aldrich) and butylated hydroxytoluene (BHT) (Thermo Fisher, Dreieich, Germany).

Finally, the screening of non-volatile substances was performed using ultra-performance liquid chromatography technology (UPLC Waters Acquity l-Class) coupled to a mass spectrometer with an orthogonal time-of-flight hybrid quadrupole analyzer (QToF-MS) with an analytical column, Cortecs UPLC C18 2.1 mm × 100 mm; 1.5 µm. To perform the semi-quantification of the identified substances, two analytical standards were injected together with the samples; for the positive detection mode (ESI+), diallyl phthalate was used, and for the negative detection mode, (ESI−) ethyl 3-(3,5-di-tert-butyl-4-hydroxyphenyl) propionate was used. 

The candidate substances detected in the different NIAS fractions were identified using specific libraries. The National Institute of Standards and Technology (NIST) library was used for volatile and semi-volatile substances, and an in-house library was used for non-volatile substances. In accordance with Osorio et al. (2019) [30], only candidate substances presenting an NIST match value above 700 were considered, whereas the mass spectra of candidate substances detected with UPLC-QTof (Waters Corporation, Milford, CT, USA) were compared with those in the internal library (mass error < 10 ppm and rt < 0.1 min). A confidence level was attributed to each candidate substance.

Finally, the toxicity of the compounds found was studied using ToxTree software [31]. This programme predicts the structural toxicity of the substances thanks to the application of different decision trees, following Cramer’s rules, or the decision tree proposed by Kroes et al. (2004) [32], which is based on the TTC (threshold of toxicological concern) approach, among others. As a result, chemical substances are classified in different categories, I, II or III, depending on their toxicity.

### 2.5. Statistical Analysis

A statistical analysis of the experimental data was performed with Statgraphics Centurion v.18.1.10 (64 bit), a commercial software. A one-way analysis of variance (ANOVA) was carried out. Differences between mean values were assessed based on confidence intervals using Tukey’s test for pairwise multiple comparisons at a *p* < 0.05 significance level.

## 3. Results and Discussion

### 3.1. Characterization of the Active Ingredient

Table 2 presents the results of the characterization of the active ingredient, orange peel extract. As can be seen, orange peel extract is rich in flavonoids and phenolic compounds, which makes it interesting to be used as an antioxidant. In fact, the antioxidant effect of phenolic and flavonoid compounds is well known, and their use in medicine has been investigated for many years [33]. However, in addition to medicine, the use of these types of phytochemicals has also been explored in other areas, such as food. In this sense, other authors have found a relationship between the content of these types of substances and the antioxidant capacity of plant extracts and agricultural by-products, giving these products characteristics that make them useful for use as antioxidant ingredients in foodstuffs [34].

### 3.2. Optical Properties

**Figure 1:**
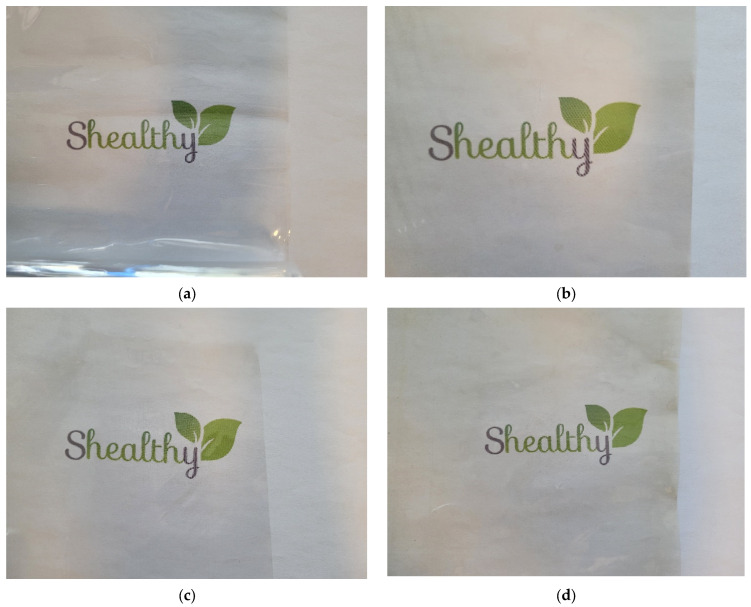
Images of the active films processed at a semi-industrial scale: (**a**) polypropylene (PP); (**b**) polypropylene + orange peel extract (PP + OPE); (**c**) polylactic acid (PLA); (**d**) polylactic acid + orange peel extract (PLA + OPE).

Figure 1 shows some images of the films processed at a semi-industrial scale. As can be seen in the photographs, all the films were transparent and showed very slight yellow coloration due to the incorporation of the orange peel extract.

**Figure 2:**
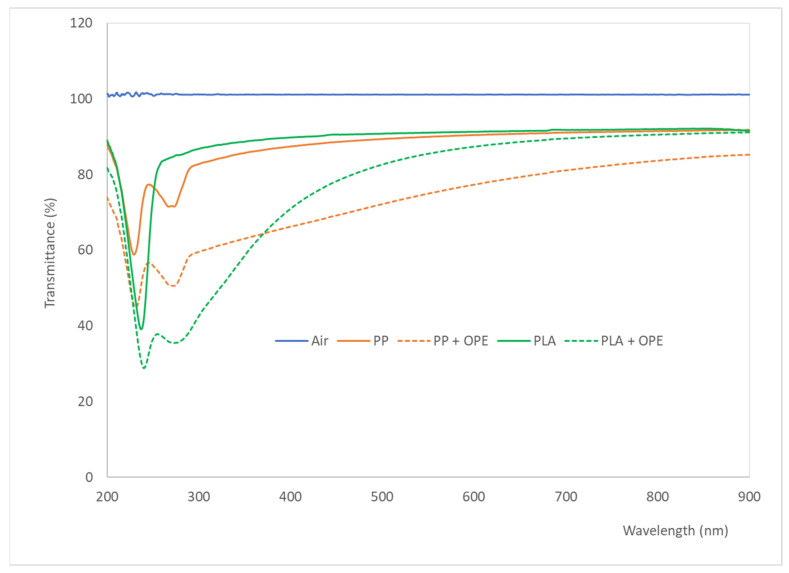
Transmittance spectra of polypropylene (PP); polypropylene + orange peel extract (PP + OPE); polylactic acid (PLA); polylactic acid + orange peel extract (PLA + OPE) films in the UV-visible range.

Figure 2 represents the percentage of transmittance of the films as a function of the wavelength. The transmittance spectrum of the air is presented as a reference. In general, the films had high transmittance in the visible range and lower transmission in the UV range. Specifically, an absorbance peak was observed around 250 nm, represented by a drop in the percentage of transmittance of 50% and 30% in PLA- and PP-based films, respectively. The observed absorption of light in the UV range may have been due to the presence of additives that acted as UV filters. In addition, in films containing the active ingredient, this decrease was accentuated. Likely, the OPE phenolic compounds with functional groups, i.e., benzoic acid, benzene, and benzoic acid methyl ester with a Pi electron, had strong potential to absorb light in the UV region to prevent UV-linked oxidation and pigment degradation [35]. As far as the visible zone is concerned, in general, all the films had high transmittance in the whole spectrum, which means that they were transparent with slight coloration, due to the low absorbance in the zone between 400 and 600 nm. Nonetheless, there was a significant difference in transmittance due to the addition of the extract in the films. For instance, at a 500 nm wavelength, the transmittance for PP and PLA films was 91% while it was only 65% and 84% for active PP and PLA films, respectively. This decrease in light transmission could have been due to the hindrance of light passage caused by the fillers distributed inside the matrix [36].

### 3.3. Thickness and Grammage

Table 3 summarizes the results of the thickness and grammage measurements of the developed films. As expected, the average thicknesses of all the films were very similar, around 70–80 µm, in line with the theoretical structure, which consisted of two layers, the external one measuring 20 µm and the internal one measuring 50 µm.

### 3.4. Mechanical Properties

Table 4 and Table 5 show the results obtained in the tensile test in the longitudinal and cross-direction, respectively. The main statistically significant differences found were related to the polymer itself. In this sense, when force was applied in both directions, the same tendency was observed; in general, PLA-based films presented higher Young’s modulus values than PP-based films, which means that they were stiffer. On the other hand, values of the elongation at break parameter were found to be about two orders of magnitude higher for PP-based films than those for PLA-based films, resulting in films that were more deformable and less brittle than PLA-based films, in line with the values obtained for Young’s modulus.

Regarding tensile strength, values of the same order were found for both types of films, although they were slightly higher for PLA-based films, which means that a higher force must be applied to deform PLA-based films compared with that required for PP-based films. In conclusion, when PP is used as the base polymer, more deformable and more flexible films are obtained than when PLA is used.

Finally, when analyzing the effect of the incorporation of active substances into the different polymeric matrices, the mechanical properties of PP were affected by the incorporation of OPE, as can be seen in the significant increase in the elongation at break and tensile strength parameters in the cross-direction test. This fact could be due to the composition of OPE, which is rich in phenolic compounds and flavonoids, which make films more deformable. This trend aligns with the results obtained by Antosik et al. (2021) [37] when incorporating green tea extract and oregano oil PP cast films. The presence of terpenes in the extract can act as a plasticizer, which can weaken the intermolecular forces between the polymeric chains, allowing an increase in elongation at break.

However, the mechanical properties evaluated in the longitudinal direction of the films developed in this study, based on PP and PLA, were modified by the incorporation of OPE.

### 3.5. Sealability

**Figure 3:**
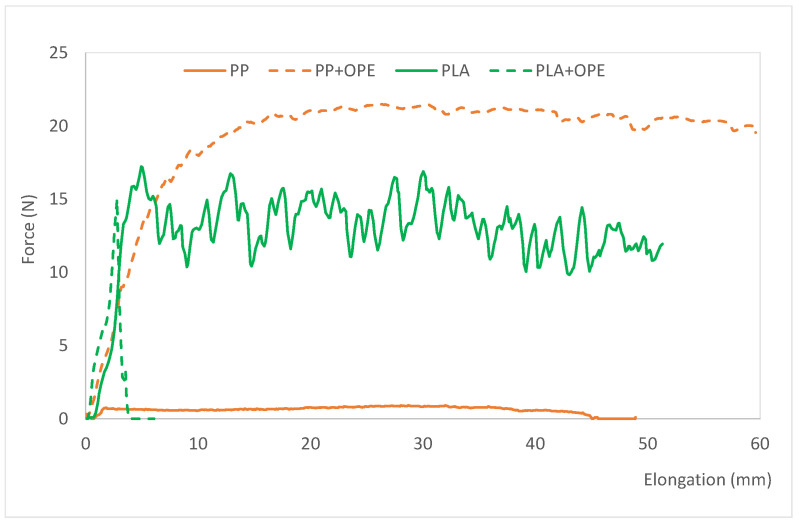
Force vs. elongation average curves of the active films, polypropylene + orange peel extract (PP + OPE) and polylactic acid + orange peel extract (PLA+ OPE), compared with those of the control films: polypropylene (PP) and polylactic acid (PLA).

Table 6 shows the values of the sealing parameters and the sealing strength of the evaluated films. Figure 3 shows the force vs. elongation plots of the films once their inner side was sealed. The sealing parameters are the combination of the temperature, pressure, and time needed to seal the film on the inner side, which is the food contact side. This test simulates the actual use of the film, conceived to make bags for fruits and vegetables, and allows us to evaluate if it is possible to manufacture the bags, since they will need to be sealed on their internal side (the food contact side). The test also allows us to evaluate the sealing strength of the seal, and therefore, whether or not the bags will withstand the weight of the packaged product without breaking at the sealing area, during transportation, storage, and opening [38].

As it can be observed, all the tested films were sealable, so bags can be made with all the references developed. No differences in the sealing parameters were observed due to the presence of the active substance, OPE. However, concerning the polymer, films based on PLA needed higher pression and a longer sealing time to be sealed than PP based films. Regarding sealing strength, this parameter abruptly increased when OPE was incorporated into the PP films. The opposite tendency was observed when using PLA as polymeric matrix. In this latter case, sealing strength was found to be significantly lower for the film containing OPE. In this sense, when OPE is incorporated into PP, structural changes occur in the polymer, perhaps due to the movement of chains, which become more available and allow stronger bonding between PP sheets, which, once sealed, are more costly to separate compared with those made with polymers without OPE. This is reflected in the higher value of the “sealing strength” parameter in the film containing OPE compared with that of the control without the active ingredient.

In any case, the results obtained show strong sealing, and the possibility of forming bags, which are expected to be suitable for fruits and vegetables from a mechanical point of view.

### 3.6. Thermal Characterization

**Figure 4:**
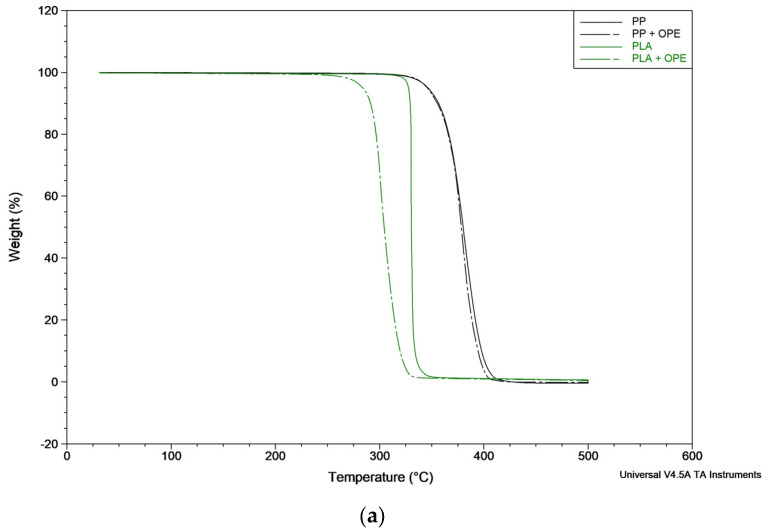

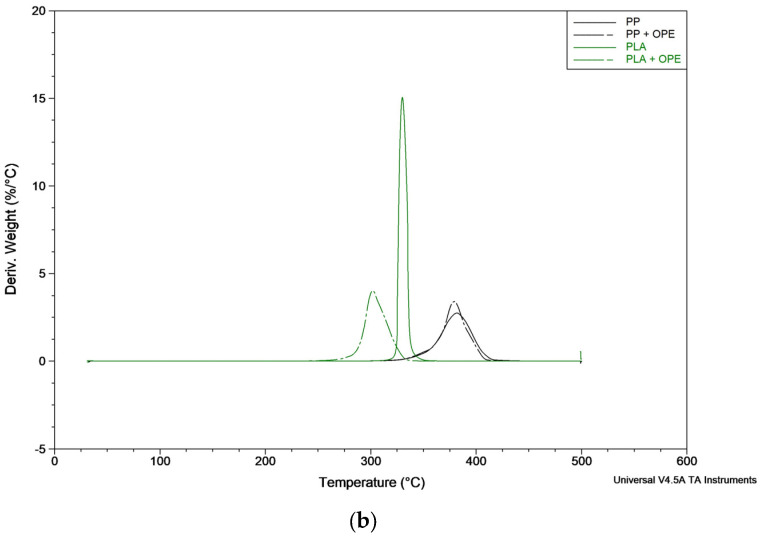
Weight loss curves (**a**) and derivative curves (**b**) from the TGA analysis of the films: polypropylene (PP); polypropylene + orange peel extract (PP + OPE); polylactic acid (PLA); and polylactic acid + orange peel extract (PLA + OPE).

The thermogram curves of the bilayer films with and without OPE in the PP and PLA matrixes are shown in Figure 4. The thermal parameters, T_dmax_, T_onset_, and residue, were analyzed and are reported in Table 7. As can be observed in the cited figure and tables, the PP bilayer decomposed in a single step, T_max_ was registered to be 383 ± 3 °C, and T_onset_ was at 365 ± 4 °C. When adding OPE to the polymer formulation, these parameters did not present variations, as the T_max_ registered was 383 ± 2 °C and the T_onset_ registered was 365 ± 3 °C. On the other hand, no residue was registered at the end of the trial.

It is well known that antioxidant compounds can increase the free volume of the polymer matrix by acting as pseudo plasticizers, and as a result, the incorporation of certain amounts of these active compounds could change the polymer’s thermal properties [39] (Lopez-Rubio and Lagaron, 2011). Nevertheless, in this study, the incorporation of OPE did not affect the thermal properties of PP, which is considered to be a positive effect as it does not significantly alter the thermal stability of the polymer. On the other hand, it could also be that OPE partially separates from the polymer phase and recrystallizes on the surface [40]. However, the other properties studied were not affected, so if this were to occur, it would not have a significant impact on the other properties of the materials obtained.

The PLA bilayer also decomposes in a single-step process with a T_max_ and T_onset_ of 330 °C; however, when adding OPE, the T_onset_ and T_max_ values decrease to 292 ± 1 °C and 302 ± 1 °C, respectively. Thus, the results obtained show that the incorporation of OPE slightly decreased the thermal stability of the PLA bilayer film. However, although there is an impact after OPE incorporation, it is not possible to quantify the extract present in the material, as it may have been degraded or lost during processing.

**Figure 5:**
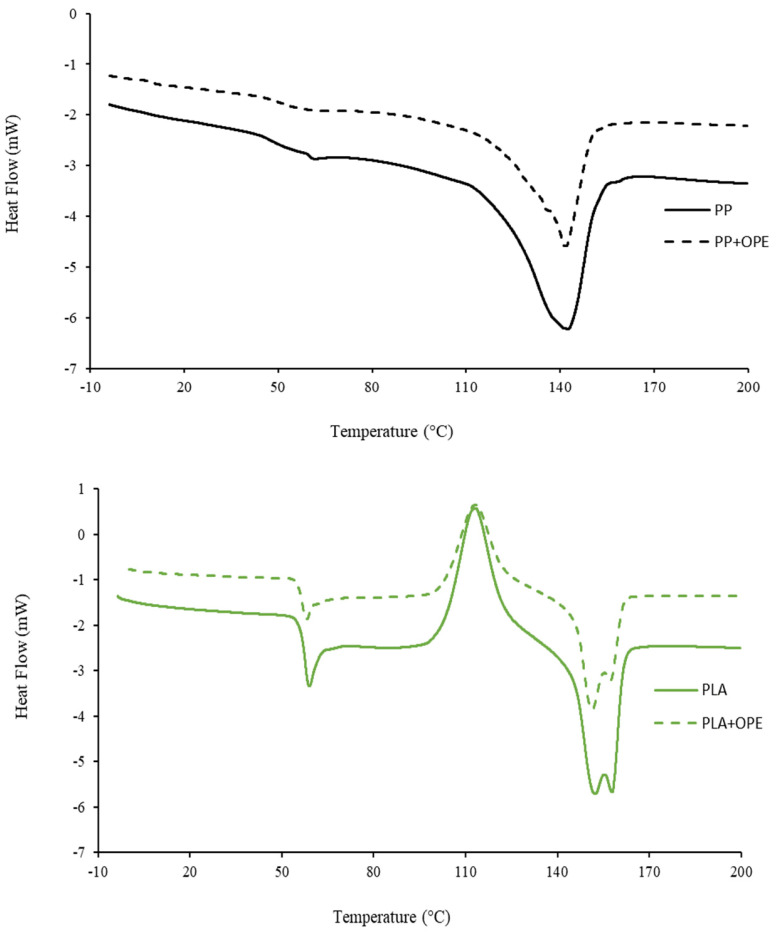
Calorimetric curves of the films: polypropylene (PP) and polypropylene + orange peel extract (PP + OPE), left; and polylactic acid (PLA) and polylactic acid + orange peel extract (PLA + OPE), right.

The calorimetric curves obtained during the heating of the samples are shown in Figure 5. The thermal parameters of the films are listed in Table 8. The PP samples exhibited a melting peak around 142 °C. A displacement of the DSC curve around 50 °C was also observed, probably due to the temperature dependence of the PP crystalline structure, while in the PLA sample curves, three different transitions could be observed: a glass transition, cold crystallization, and melting. Both PLA and PLA + OPE showed cold crystallization and melting at approximately 114 °C and 150 °C, respectively, indicating that OPE did not modify the mobility of the polymer chains. The extracts did not influence the thermal properties of the films in the studied temperature range, since only polymer transitions were observed.

### 3.7. Antimicrobial Activity

#### 3.7.1. Antimicrobial Activity in the Vapor Phase

**Figure 6:**
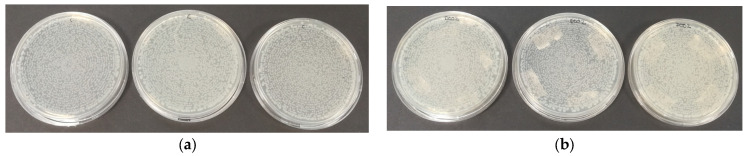
Plates inoculated with *E. coli* (24 h at 36 °C). Antimicrobial test in the vapor phase: (**a**) positive control; (**b**) PP + OPE.

**Figure 7:**
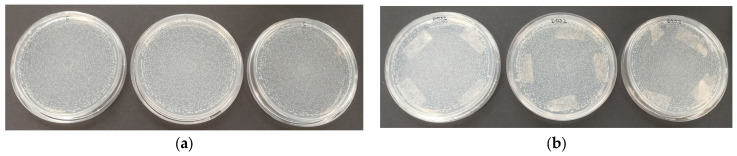
Plates inoculated with *L. innocua* (24 h at 36 °C). Antimicrobial test in the vapor phase: (**a**) positive control; (**b**) PP + OPE.

**Figure 8:**
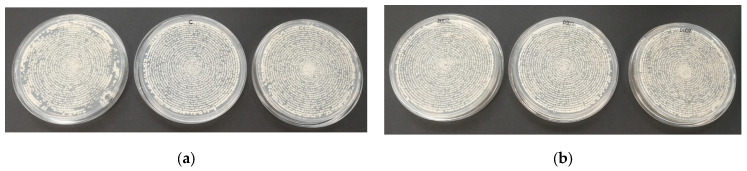
Plates inoculated with *S. cerevisiae* (3 days at 25 °C). Antimicrobial test in the vapor phase: (**a**) positive control; (**b**) PP + OPE.

**Figure 9:**
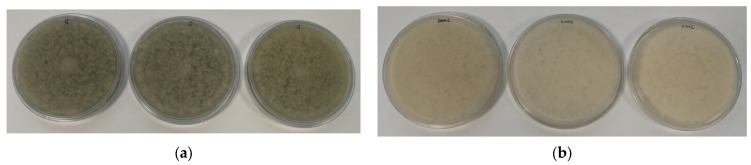
Plates inoculated with *A. niger* (5 days at 25 °C). Antimicrobial test in the vapor phase: (**a**) positive control; (**b**) PP + OPE.

Figure 6, Figure 7, Figure 8 and Figure 9 show photographs of the Petri dishes after the antimicrobial activity test in the vapor phase of positive controls and PP + OPE samples. Photographs of the positive controls are also shown, to be compared with, since they are a reference for the growth of each one of the selected microorganisms. As the same results were achieved with all the samples tested, i.e., PP, PP + OPE, PLA and PLA + OPE, only PP + OPE photographs are shown, as an illustration of the results of the tests.

In this sense, when comparing the inoculated plates containing the test specimens, in all cases (every reference and every microorganism) the same degree of growth was observed, with no inhibition zones in any case. Due to this fact, it can be verified that none of the references analyzed presented antimicrobial activity in the vapor phase against Gram-positive bacteria, Gram-negative bacteria, fungi, or yeasts. This may be due to the fact that the active components of the orange peel extract are not volatile or have low volatility and therefore do not have the ability to reach the surface of the inoculated Petri dish through the headspace to exhibit antimicrobial activity against any of the types of microorganisms tested. Otherwise, it may be becausee the amount reaching the surface is insufficient to create such an effect.

#### 3.7.2. Antimicrobial Activity by Direct Contact

**Figure 10:**
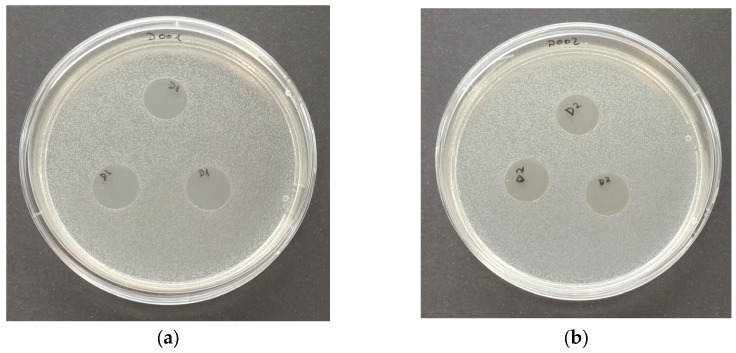
Plates inoculated with *E. coli* (24 h at 36 °C). Antimicrobial test in the contact phase: (**a**) PP; (**b**) PP + OPE.

**Figure 11:**
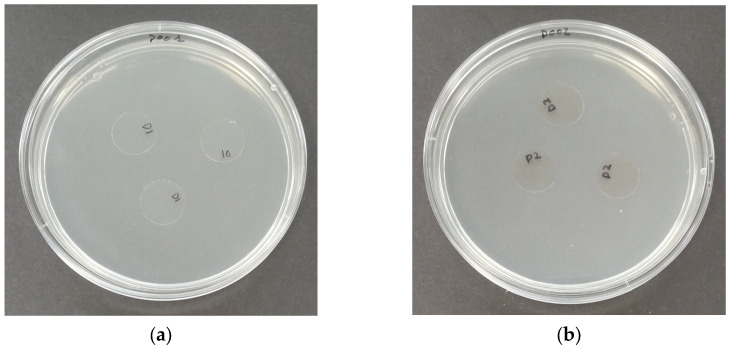
Plates inoculated with *L. innocua* (24 h at 36 °C). Antimicrobial test in the contact phase: (**a**) PP; (**b**) PP + OPE.

**Figure 12:**
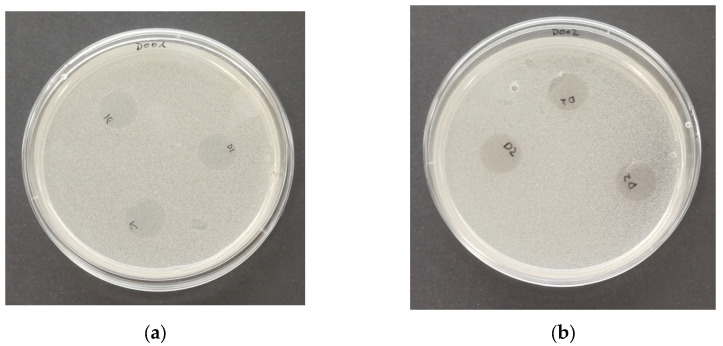
Plates inoculated with *S. cerevisiae* (3 days at 25 °C). Antimicrobial test in the contact phase: (**a**) PP; (**b**) PP + OPE.

**Figure 13:**
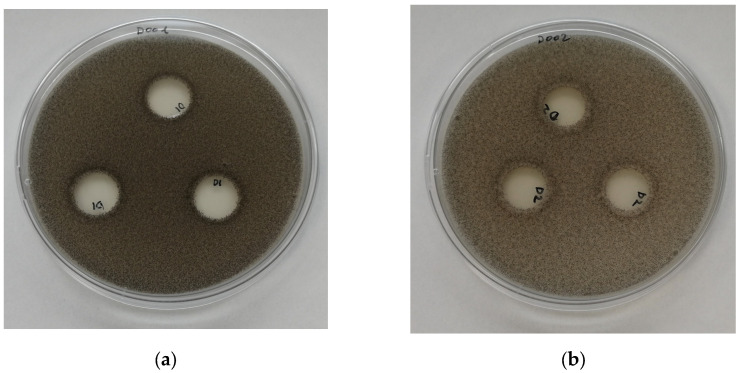
Plates inoculated with A. niger (5 days at 25 °C). Antimicrobial test in the contact phase: (**a**) PP; (**b**) PP + OPE.

Figure 10, Figure 11, Figure 12 and Figure 13 show images of the Petri dishes inoculated, put into contact with the film specimens and incubated in the conditions required for each one of the microorganisms selected to test the antimicrobial activity via contact. As same results were achieved with all the samples tested, i.e., PP, PP + OPE, PLA and PLA + OPE, only PP and PP + OPE photographs are shown, as an illustration of the results of the tests.

In all the references and for all the microorganisms analyzed, the absence of an inhibition zone was observed, which literally means that there was no transfer of water-soluble antimicrobial substances; consequently, this can be interpreted as the absence of antimicrobial activity in the direct contact of the films analyzed against Gram-positive bacteria, Gram-negative bacteria, fungi, or yeasts. In this case, it is possible that the active substances present in the orange peel extract did not migrate in a sufficient quantity to exhibit significant antimicrobial activity against the types of microorganisms selected for this study.

### 3.8. Antioxidant Activity

**Figure 14:**
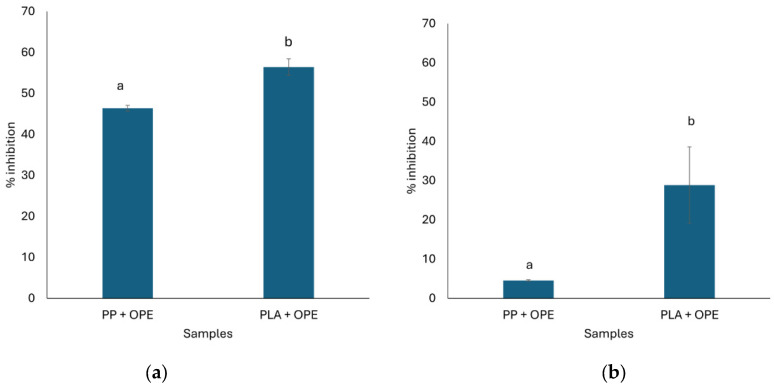
Antioxidant activity of active film samples, polypropylene + orange peel extract (PP + OPE) and polylactic acid + orange peel extract (PLA + OPE), expressed as inhibition percentage (%), (**a**) with extraction and (**b**) with direct contact. Different lowercase letters means that samples are statistically different (*p* < 0.05).

The DPPH radical scavenging activity of the films was evaluated via the extraction and direct contact of DPPH radicals with the film samples (Figure 14a,b). The antioxidant activity of the plant extract is mainly caused by its phenolic compounds, which involve an electron transfer reaction, which ultimately causes a reduction in DPPH molecules, as reported by Chen et al. (2017) [41] for orange peel extract. Overall, the extraction method yielded higher average antioxidant capacity from the films as compared with the direct contact method. On the other hand, active PLA films displayed significantly higher antioxidant capacity (28–56%) via both methods as compared with active PP films (4–46%), which could be due to the lower compatibility between the extract and PLA, which allowed for higher release of antioxidants from the PLA-based films [42]. It has been reported that hydroxyl groups in quinic acid present in orange peel extract can provide radical scavenging activity [43]. Numerous aspects, such as the films’ physical and chemical characteristics and the antioxidant compounds’ nature, can be attributed to the varying antioxidant activity between the direct extraction and direct contact approaches for measuring antioxidant capacity.

The polymer films’ composition and structure have a major impact on how antioxidant agents are released. For example, PLA films are more compatible with phenolic compounds than PP films are because of their intrinsic polarity, which allows for the more effective release of antioxidants. This was corroborated by the findings of Fabra et al. (2016) [44], who showed the importance of polymer matrix characteristics in influencing the diffusion and release of active/bioactive components. In addition, antioxidants can be solubilized to a large extent via the direct extraction method based on solvent interaction, which could lead to a higher release rate. In this regard, the study by Espitia et al. (2014) [45] supports this idea, emphasizing how solvent characteristics can alter the extraction efficiency of active chemicals from edible films.

### 3.9. Food Safety Assessment

Migration tests

Table 9 shows a summary of the results obtained in the overall migration test carried out on the active packaging prototypes. The results are expressed as the average value ± the uncertainty of the method, for each of the analyzed simulants. As can be seen, all the overall migration values obtained are lower than the established limit of 10 mg/dm^2^, so all the prototypes are compliant. In this sense, based on the overall migration test result alone, the films would be suitable for contact with fresh fruits and vegetables as well as washed and cut (ready to eat) fruits and vegetables during prolonged storage at room temperature or below, including packaging under hot-fill conditions and/or heating up to a temperature, T, of 70 °C ≤ T ≤ 100 °C for a maximum of t = 120/2 ^ [(T − 70)/10] minutes.

On the other hand, the results of the specific migration of the different substances analyzed are shown in the following tables. Table 10 presents the specific migration results for primary aromatic amines, whereas Table 11 shows the results of the specific migration of metals included in Annex II of the Plastics Regulation. The results are expressed as the average value ± uncertainty of the method, for each of the analyzed simulants.

As far as compliance with the restrictions laid down in Annex II of the Plastics Regulation for primary aromatic amines is concerned, all the references are compliant, as the specific migration of each one of these compounds is, in all cases, below the detection limit, and so it is below the migration limit established for these substances individually in 2 ppb.

Regarding metals, the specific migration of the metals analyzed are in acordance with the limits set out by legislation in all the references, most of them even being below their limit of quantification.

In this respect, if compliance is only be based on this analysis, the films would be suitable for contact with fresh fruits and vegetables as well as washed and cut (ready to eat) fruits and vegetables for a maximum of 30 days at temperatures up to 40 °C. These conditions include the foreseeable use of the films.

Screening of NIAS

The substances identified in the different fractions are presented in the following tables. Table 12 shows the list of substances in the volatile fraction. No substances were found for this fraction of compounds coming from the reference, PP + OPE. Table 13 displays the list of compounds identified in the semi-volatile fraction of the NIAS. Semi-volatile substances were only identified in the PP reference in simulant A and in the PLA reference in both simulants A and B. Finally, in Table 14, results of the screening analysis of the non-volatile fraction of NIAS are shown.

In the screening of the different fractions of NIAS, in the case of the PP film sample, four substances in total were detected, from which ethanol and triacetin were detected in the volatile fraction, while one semi-volatile substance (2-fluoroacetamide) and one non-volatile substance, which was galactitol, were detected.

When adding OPE into the PP matrix, no volatile or semi-volatile substances were detected, but five non-volatile substances were identified. The incorporation of OPE resulted in the release of substances clearly coming from OPE like ethylvanillin. Other substances such as Cyasorb 2908, bisphenol A, and methylparaben may come from additives and impurities in raw materials; however, they were not detected in the PP film. This could be due to the fact that the incorporation of OPE could have opened up the chains of the polymer, facilitating the mobility of the molecules and their diffusion through the PP matrix into the migrating extract.

On the other hand, from PLA film, substances from polymer degradation, residues of monomers, and starting substances, as well as impurities that came with the raw materials used in the processing of PLA-based films were found. In this sense, five volatile substances (triacetin, 2-hydroxyethylhydrazine, allyl acetate, acetic anhydride, and lactide), one semi-volatile substance (sulfur dichloride), and six non-volatile substances, 2,7-dichlorofluorescin diacetate, 2-amino-4-methylbenzophenone, 3,3′-diclorobenzidine, epigallocatechin gallate, triglycerol, and 6-hydroxycaproic acid, were detected.

On the other hand, from PLA + OPE film, in total, 14 volatile substances, 2-hydroxyethylhydrazine, acetic anhydride, lactide, diacetyl suplhide, acetic acid, 2,3-butanedione, 2,3-pentanedione, cyclobutylamine, (R,R)-3-Chloro-2-butanol, [S-(R,R)]-2,3-butanediol, 2-hydroxy-3-pentanone, isobutane, 4-oxo-pentanoci acid, and ethyl pyruvate, and 13 non-volatile substances, ethylvanillin, methylparaben, 2,7-dichlorofluorescin diacetate, 3,3′-dichlorobenzidine, epigallocatechin gallate, triglycerol, 6-hydroxycaproic acid, Cyasorb UV 12, naringin, succinic acid, adipic acid, and trans-ferulic acid, were detected. Furthermore, the presence of compounds such as ethylvanillin and naringin indicated the incorporation of OPE into the PLA matrix.

## 4. Conclusions

The results of this study show that the incorporation of OPE into PP and PLA is feasible, and therefore, the processability of active packaging via semi-industrial-scale extrusion techniques is feasible. However, the retention rate of OPE is unknown, and further studies should be carried out. The films obtained were transparent with slight coloration when OPE was added. The mechanical properties of the active films were affected by OPE, as the elongation at break and tensile strength increased in the transverse direction of the PP films. However, the main differences found were related to the polymer itself rather than to the presence of OPE. The seal strength improved when OPE was incorporated into the PP matrix. The opposite trend was observed in the PLA matrix, and this could have been due to the lower affinity of OPE to PLA than to PP. However, both active films (PP + OPE and PLA + OPE) were sealable. Thus, it was verified that both active materials can be used for the manufacture of packaging materials, e.g., flow pack bags for fruit and vegetable packaging. In relation to the functionality of the films developed, no antimicrobial activity was observed in the in vitro studies against *L. innocua*, *S. cerevisiae*, *A. niger*, and *E. coli*. However, the films showed antioxidant activity in in vitro tests with the DPPH radical. Therefore, a possible application for the active films developed in this work is the packaging of oxidation-sensitive products.

## Figures and Tables

**Table 1:** Detail of the structure and composition of references of active film manufactured at a semi-industrial scale: polypropylene (PP); polypropylene + orange peel extract (PP + OPE); polylactic acid (PLA); polylactic acid + orange peel extract (PLA + OPE).

Sample Code	Base Polymer	Concentration of Active Ingredient (% (*w*/*w*))	Structure (Polymeric Layer and Thickness)
PP	PP	---	PP 20 µm/PP 50 µm
PP + OPE	PP	10	PP 20 µm/PP + 10% OPE 50 µm
PLA	PLA	---	PLA 20 µm/PLA 50 µm
PLA + OPE	PLA	7.5	PLA 20 µm/PLA + 7.5% OPE 50 µm

**Table 2:** Composition of the active ingredient, orange peel extract (OPE) (mean ± SD).

Compound	µg/mL Extract
2-(E)-O-Feruloyl-D-galactaric acid isomer I	525.50 ± 3.39
Caffeic acid 3-O-glucuronide isomer I	212.98 ± 1.45
2-(E)-O-Feruloyl-D-galactaric acid isomer II	333.98 ± 2.20
Caffeic acid 3-O-glucuronide isomer II	200.78 ± 1.37
Cynaroside A	15.88 ± 0.31
Caffeoylglycolic acid methyl ester	59.40 ± 0.49
Sinapinic acid-O-glucuronide	400.02 ± 2.61
Rutoside	3.88 ± 0.08
Feruloyl isocitric acid	80.74 ± 0.63
Apigenin 6,8di-C-glucoside (Vicenin-2)	42.23 ± 0.82
Prunin	46.65 ± 0.91
Isorhamnetin-3-O-rutinoside isomer I	12.98 ± 0.25
Caffeoylmalic acid isomer I	81.71 ± 0.63
Isorhamnetin-3-O-rutinoside isomer II	7.06 ± 0.14
Quercitrin	0.49 ± 0.01
Alpha-Glucosyl Hesperidin	4.47 ± 0.09
Vitexin-O-pentoside isomer I	4.35 ± 0.08
Vitexin-O-pentoside isomer II	3.49 ± 0.07
Naringin hydrate	2.51 ± 0.05
Naringenin	10.90 ± 0.21
Caffeoylmalic acid isomer II	33.71 ± 0.33
Narirutin isomer I	23.57 ± 0.46
Eriocitrin	3.90 ± 0.08
Narirutin isomer II	28.41 ± 0.55
Hesperetin	4.83 ± 0.09
Hesperidin	32.88 ± 0.64
Kaempferol 3-[2″-glucosyl-6″-acetyl-galactoside]7-glucoside	2.83 ± 0.06
Isorhamnetin-3-O-rutinoside isomer III	2.70 ± 0.05
Apigenin 7-O-neohesperidoside	11.42 ± 0.22
Isosakuranetin	4.89 ± 0.10
Didymin	1.08 ± 0.02
3′,4′-Didemethylnobiletin	2.08 ± 0.04
Sum of phenolic acids	1928.83 ± 13.11
Sum of flavonoids	273.49 ± 5.34
Sum of phenolic compounds	2202.32 ± 18.45

**Table 3:** Results obtained for the thickness and grammage (mean ± SD, n = 10) measurements of the films developed: polypropylene (PP); polypropylene + orange peel extract (PP + OPE); polylactic acid (PLA); polylactic acid + orange peel extract (PLA + OPE). The same superscripts within the same column indicate no statistically significant different values (*p* < 0.05).

Reference	Thickness (mm)	Grammage (g·m^−2^)
PP	0.078 ± 0.001 ^a^	71 ± 1 ^a^
PP + OPE	0.077 ± 0.002 ^a^	69 ± 1 ^a^
PLA	0.075 ± 0.005 ^a^	85 ± 3 ^b^
PLA + OPE	0.078 ± 0.010 ^a^	86 ± 5 ^b^

**Table 4:** Mechanical parameters. Young’s modulus (GPa), elongation at break (%), and tensile strength (MPa) in the longitudinal direction of the films: polypropylene (PP); polypropylene + orange peel extract (PP + OPE); polylactic acid (PLA); and polylactic acid + orange peel extract (PLA + OPE). (mean ± SD, n = 6). The same superscripts within the same column indicate no statistically significant different values (*p* < 0.05).

Reference	Young’s Modulus (GPa)	Elongation at Break (%)	Tensile Strength (MPa)
PP	0.68 ± 0.04 ^a^	696 ± 64 ^a^	31 ± 4 ^a^
PP + OPE	0.68 ± 0.03 ^a^	644 ± 51 ^a^	31 ± 4 ^a^
PLA	2.36 ± 0.13 ^b^	3.47 ± 0.4 ^b^	58 ± 4 ^b^
PLA + OPE	2.2 ± 0.2 ^b^	3.13 ± 0.33 ^b^	52 ± 3 ^b^

**Table 5:** Mechanical parameters. Young’s modulus (GPa), elongation at break (%), and tensile strength (MPa) in the cross-direction of the films: polypropylene (PP); polypropylene + orange peel extract (PP + OPE); polylactic acid (PLA); and polylactic acid + orange peel extract (PLA + OPE). (Mean ± SD, n = 6). The same superscripts within the same column indicate no statistically significant different values (*p* < 0.05).

Reference	Young’s Modulus (GPa)	Elongation at Break (%)	Tensile Strength (MPa)
PP	0.63 ± 0.03 ^a^	696 ± 41 ^a^	25 ± 2 ^a^
PP + OPE	0.64 ± 0.05 ^a^	796 ± 91 ^b^	32 ± 5 ^b^
PLA	2.42 ± 0.09 ^b^	2.54 ± 0.26 ^c^	50 ± 3 ^c^
PLA + OPE	2.47 ± 0.13 ^b^	2.45 ± 0.2 ^c^	50 ± 2 ^c^

**Table 6:** Results obtained for the sealing strength, expressed as force peak (N) and average force (N), and for the sealing parameters, i.e., the temperature (°C), pressure (Psi), and time (s) of the films: polypropylene (PP); polypropylene + orange peel extract (PP + OPE); polylactic acid (PLA); and polylactic acid + orange peel extract (PLA + OPE). (mean ± SD, n = 6). The same superscripts within the same column indicate no statistically significant different values (*p* < 0.05).

Reference	Sealing Parameters			Sealing Strength	
	T (°C)	P (Psi)	Time (s)	Max Force (N)	Average Force (N)
PP	130	38.5	0.8	1.0 ± 0.4 ^a^	0.7 ± 0.3 ^a^
PP + OPE	130	38.5	0.6	21.4 ± 0.5 ^b^	19 ± 1 ^b^
PLA	130	40	1	19.7 ± 1.9 ^c^	12 ± 3 ^c^
PLA + OPE	125	40	1	14.4 ± 0.8 ^d^	No data *

* It was not possible to obtain an average value because the material broke just after the peak of the maximum force (see Figure 3).

**Table 7:** Thermal properties extracted from the TGA analysis of the films: polypropylene (PP); polypropylene + orange peel extract (PP + OPE); polylactic acid (PLA); and polylactic acid + orange peel extract (PLA + OPE). (mean ± SD, n = 6). The same superscripts within the same column indicate no statistically significant different values (*p* < 0.05).

Reference	T_onset_ (°C)	T_max_ (°C)	Residue
PP	362 ± 0 ^c^	383 ± 3 ^c^	No ^a^
PP + OPE	365 ± 3 ^c^	383 ± 2 ^c^	No ^a^
PLA	330 ± 0 ^a^	330 ± 0 ^a^	0.6 ± 0.1 ^b^
PLA + OPE	292 ± 1 ^b^	302 ± 1 ^b^	0.5 ± 0.1 ^b^

**Table 8:** DSC results of PP, PLA, and active films (PP + OPE; PLA + OPE).

	Glass Transition			Crystallization				Melting			
Reference	T_On-set_ (°C)	T_End-set_ (°C)	T_inflection point_ (°C)	T_On-set_ (°C)	T_End-set_ (°C)	T_pk_ (°C)	∆H (J/g)	T_On-set_ (°C)	T_End-set_ (°C)	T_pk_ (°C)	∆H (J/g)
PP	-	-	-	-	-	-	-	68	167	142	62
PP + OPE	-	-	-	-	-	-	-	68	167	142	65
PLA	56	56	57	87	136	113	39	163	174	152	38
PLA + OPE	55	57	57	86	136	114	30	136	170	151	28

**Table 9:** Results of the overall migration tests (mg/dm^2^) on the films: polypropylene (PP); polypropylene + orange peel extract (PP + OPE); polylactic acid (PLA); and polylactic acid + orange peel extract (PLA + OPE).

Reference	A (Ethanol 10% (*v*/*v*))	B (Acetic Acid 3% (*w*/*v*))
PP	0.6 ± 1	0.2 ± 3.0
PP + OPE	0.1 ± 1	5.1 ± 3.7
PLA	0.4 ± 1.2	0.8 ± 3.2
PLA + OPE	0.6 ± 1.7	0.8 ± 3.2

**Table 10:** Results of the specific migration tests on the primary aromatic amines of the films: polypropylene (PP); polypropylene + orange peel extract (PP + OPE); polylactic acid (PLA); and polylactic acid + orange peel extract (PLA + OPE).

		Average Result (µg/kg)			
Substance	DL (µg/kg)	PP	PP + OPE	PLA	PLA + OPE
o-Tolidine	2	n.d.	n.d.	n.d.	n.d.
1,4-benzenediamine	2	n.d.	n.d.	n.d.	n.d.
o-Toluidine	2	n.d.	n.d.	n.d.	n.d.
Aniline	2	n.d.	n.d.	n.d.	n.d.
4-chloroaniline	2	n.d.	n.d.	n.d.	n.d.
4-aminoazotoluene	1	n.d.	n.d.	n.d.	n.d.
2,4-diaminotoluene	1	n.d.	n.d.	n.d.	n.d.
4,4′-oxydianiline	1	n.d.	n.d.	n.d.	n.d.
2-methyl-5-nitroaniline	1	n.d.	n.d.	n.d.	n.d.
4,4-diaminodifenylmethane	1	n.d.	n.d.	n.d.	n.d.
4-aminoazobencene	1	n.d.	n.d.	n.d.	n.d.
Biphenyl-4-ylamine	1	n.d.	n.d.	n.d.	n.d.
o-dianisidine	1	n.d.	n.d.	n.d.	n.d.
4-methyl-3-nitroaniline	1	n.d.	n.d.	n.d.	n.d.
2-naphtylamine	1	n.d.	n.d.	n.d.	n.d.
4-chloromethylaniline	2	n.d.	n.d.	n.d.	n.d.
4,4-methylene-bis(2-mehylaniline)	1	n.d.	n.d.	n.d.	n.d.
Benzidine	1	n.d.	n.d.	n.d.	n.d.
2-methoxy-5-mehylaniline	1	n.d.	n.d.	n.d.	n.d.
4,4’-Thyodianiline	1	n.d.	n.d.	n.d.	n.d.
2,4-diaminoanisole	2	n.d.	n.d.	n.d.	n.d.
o-anisidine	1	n.d.	n.d.	n.d.	n.d.
2,6-dimethylaniline	1	n.d.	n.d.	n.d.	n.d.
2,6-diaminotoluene	1	n.d.	n.d.	n.d.	n.d.
2,4-dimethylaniline	1	n.d.	n.d.	n.d.	n.d.
4,4-methylen-bis(2-chloroaniline)	1	n.d.	n.d.	n.d.	n.d.
1-naphthylamine	1	n.d.	n.d.	n.d.	n.d.
3,3′-dichlorobenzidine	2	n.d.	n.d.	n.d.	n.d.

n.d.: not detected; DL: detection limit.

**Table 11:** Results of the specific migration tests. Metals. The values in brackets represent the limit of quantification (LQ, expressed as mg/kg) of the films: polypropylene (PP); polypropylene + orange peel extract (PP + OPE); polylactic acid (PLA); and polylactic acid + orange peel extract (PLA + OPE).

		Average Result (mg/kg) ± Uncertainty			
Substance	SML (mg/kg) ^(1)^	PP	PP + OPE	PLA	PLA + OPE
Li	0.6	<LQ (0.05)	<LQ (0.05)	<LQ (0.05)	<LQ (0.05)
Na ^(2)^	Not specified	<LQ (0.5)	<LQ (0.5)	<LQ (0.5)	<LQ (0.5)
Mg ^(2)^	Not specified	<LQ (0.05)	<LQ (0.05)	0.22 ± 0.05	0.22 ± 0.05
Al	1	<LQ (0.05)	<LQ (0.05)	<LQ (0.05)	<LQ (0.05)
K ^(2)^	Not specified	<LQ (0.1)	<LQ (0.1)	<LQ (0.1)	<LQ (0.1)
Ca ^(2)^	Not specified	<LQ (0.1)	<LQ (0.1)	<LQ (0.1)	<LQ (0.1)
Cr	0.01	<LQ (0.005)	0.006 ± 0.001	0.006 ± 0.001	<LQ (0.005)
Mn	0.6	<LQ (0.005)	<LQ (0.005)	<LQ (0.005)	<LQ (0.005)
Fe	48	<LQ (1)	<LQ (1)	<LQ (1)	<LQ (1)
Co	0.05	<LQ (0.005)	<LQ (0.005)	<LQ (0.005)	<LQ (0.005)
Ni	0.02	0.004 ± 0.001	<LQ (0.001)	<LQ (0.001)	<LQ (0.001)
Cu	5	<LQ (0.05)	<LQ (0.05)	<LQ (0.05)	<LQ (0.05)
Zn	5	<LQ (0.5)	<LQ (0.5)	<LQ (0.5)	<LQ (0.5)
Cd	0.002	<LQ (0.001)	<LQ (0.001)	<LQ (0.001)	<LQ (0.001)
Ba	1	<LQ (0.005)	<LQ (0.005)	<LQ (0.005)	<LQ (0.005)
Pb	0.01	<LQ (0.005)	<LQ (0.005)	<LQ (0.005)	<LQ (0.005)
Hg	0.01	<LQ (0.0001)	<LQ (0.0001)	<LQ (0.0001)	<LQ (0.0001)
As	0.01	<LQ (0.001)	<LQ (0.001)	<LQ (0.001)	<LQ (0.001)
Sb	0.04	<LQ (0.01)	<LQ (0.01)	<LQ (0.01)	<LQ (0.01)
La	0.050	<LQ (0.01)	<LQ (0.01)	<LQ (0.01)	<LQ (0.01)
Eu	0.050	<LQ (0.005)	<LQ (0.005)	<LQ (0.005)	<LQ (0.005)
Gd	0.050	<LQ (0.001)	<LQ (0.001)	<LQ (0.001)	<LQ (0.001)
Tb	0.050	<LQ (0.005)	<LQ (0.005)	<LQ (0.005)	<LQ (0.05)

^(1)^ SML: specific migration limit under Regulation (EU) No 10/2011; ^(2)^ restrictions under Regulation (EC) No 1333/2008 and Regulation (EC) No 1334/2008 on food additives and food flavorings.

**Table 12:** List of substances identified in the volatile fraction of NIAS from the films: polypropylene (PP); polypropylene + orange peel extract (PP + OPE); polylactic acid (PLA); and polylactic acid + orange peel extract (PLA + OPE).

Substance Name	CAS	Reference	Confidence Level	TTC	Concentration (mg/kg) ^(1)^	Worst Case Migration (mg/kg) ^(2)^
Ethanol	64-17-5	PP	80%	l	172	0.73
Triacetin	102-76-1	PP	80%	l	5705	24.14
		PLA	70%		271	1.39
2-hydroxyethylhydrazine	109-84-2	PLA	80%	lll	87	0.45
		PLA + OPE	80%		131	0.68
Allyl acetate	591-87-7	PLA	70%	ll	75	0.38
Acetic anhydride	108-24-7	PLA	80%	lll	<37	<0.16
		PLA + OPE	80%		356	1.84
Lactide	95-96-5	PLA	80%	l	530	2.72
		PLA + OPE	70%		754	3.89
Diacetyl sulphide	3232-39-1	PLA + OPE	80%	(*)	153	0.79
Acetic acid	64-19-7	PLA + OPE	70%	l	819	4.23
2,3-butanedione	431-03-8	PLA + OPE	80%	lll	132	0.68
2,3-pentanedione	600-14-6	PLA + OPE	80%	lll	253	1.30
Cyclobutylamine	2516-34-9	PLA + OPE	80%	lll	73	0.38
(R,R)-3-Chloro-2-butanol	10325-40-3	PLA + OPE	70%	(*)	79	0.41
[S-(R,R)]-2,3-butanediol	19132-06-0	PLA + OPE	80%	(*)	1659	8.56
2-hydroxy-3-pentanone	5704-20-1	PLA + OPE	70%	(*)	<37	<0.16
Isobutane	75-28-5	PLA + OPE	80%	l	17	0.09
4-oxopentanoic acid	123-76-2	PLA + OPE	70%	l	96	0.50
Ethyl pyruvate	617-35-6	PLA + OPE	80%	l	303	1.56

(*) Cramer classification not available in ToxTree; ^(1)^ Concentration of the compound in the material obtained via comparison with the semi-quantified analytical standard; ^(2)^ migration estimated considering that 100% of the amount of the substance semi-quantified migrates to the food and taking into account the standard convention of 6 dm^2^ of material.

**Table 13:** List of substances identified in the semi-volatile fraction of NIAS from the films: polypropylene (PP); polypropylene + orange peel extract (PP + OPE); polylactic acid (PLA); and polylactic acid + orange peel extract (PLA + OPE).

Substance Name	CAS	Reference	Simulant	Confidence Level	TTC	Migration (BHT) (mg/kg) ^(1)^	Migration (Benzophenone) (mg/kg) ^(1)^
2-fluoroacetamide	640-19-7	PP	A	80%	lll	<0.996	0.795
Sulfur dichloride	10545-99-0	PLA	A	80%	(*)	<0.996	0.77
			B	80%		<0.996	0.77

(*) Cramer classification not available in ToxTree; ^(1)^ migration estimated considering the standard convention that 6 dm^2^ of material is in contact with 1 kg of food and based on the concentration values obtained via comparison with the semi-quantified analytical standard indicated in parentheses.

**Table 14:** List of substances identified in the non-volatile fraction of NIAS from the films: polypropylene (PP); polypropylene + orange peel extract (PP + OPE); polylactic acid (PLA); and polylactic acid + orange peel extract (PLA + OPE).

Substance Name	CAS	Reference	Simulant—ESI Mode	Confidence Level	TTC	Migration (mg/kg) ^(1)^
Galactitol	608-66-2	PP	A—ESI+	60%	l	0.0103
Cyasorb 2908	67845-93-6	PP + OPE	A—ESI+	100%	(*)	0.00726
tri-n-butyl acetyl citrate	77-90-7	PP + OPE	A—ESI+	100%	(*)	0.03696
Ethylvanillin	121-32-4	PP + OPE	A—ESI−	100%	l	0.00777
		PLA + OPE	A—ESI−	100%		0.01410
Bisphenol A	80-05-7	PP + OPE	A—ESI−	100%	lll	0.01998
4-hydroxybenzoic acid, methyl ester (methylparaben)	99-76-3	PP + OPE	A—ESI−	100%	l	0.00928
		PLA + OPE	A—ESI−	100%		0.02003
2,7-dichlorofluorescin diacetate	4091-99-0	PLA	A—ESI+	100%	(*)	0.01358
		PLA + OPE	A—ESI+	100%		0.010498
2-amino-4-methylbenzophenone	36192-63-9	PLA	A—ESI+	100%	(*)	0.002964
3,3′-diclorobenzidine	91-94-1	PLA	A—ESI+	100%	lll	0.008446
		PLA + OPE	A—ESI+	100%		0.006766
epigalloecatechin gallate	989-51-5	PLA	A—ESI+	100%	lll	0.003665
		PLA + OPE	A—ESI+	100%		0.003295
triglycerol	20411-31-8, 56090-54-1	PLA	A—ESI+	100%	lll	0.111809
		PLA + OPE	A—ESI+	100%		0.077649
6-hydroxycaproic acid	1191-25-9	PLA	A—ESI−	100%	(*)	0.032810
		PLA + OPE	A—ESI−	100%		0.032051
cyasorb uv 12 (2,2′-dihydroxy-4,4′-dimethoxybenzophenone)	131-54-4	PLA + OPE	A—ESI+	100%	lll	0.003685
Naringin	10236-47-2	PLA + OPE	A—ESI−	100%	(*)	0.038564
Succinic acid	110-15-6	PLA + OPE	A—ESI−	100%	l	0.057728
Adipic acid	124-04-9	PLA + OPE	A—ESI−	100%	l	0.009255
trans-ferulic acid ((E)-4′-hydroxy-3′-methoxycinnamic acid)	537-98-4	PLA + OPE	A—ESI−	100%	(*)	0.051075

(*) Cramer classification not available in ToxTree; ^(1)^ migration estimated considering the standard convention that 6 dm^2^ of material is in contact with 1 kg of food and based on the concentration values obtained via comparison with the semi-quantified analytical standard indicated in parentheses.

## Data Availability

The results in this article are available on request from the corresponding author due to there are no additional data relevant to the understanding of the results beyond what is specified in the article.
